# Podcasts as a Method to Deliver Education on Stigma Surrounding Opioid Use Disorder

**DOI:** 10.3390/pharmacy10060161

**Published:** 2022-11-29

**Authors:** Logan M. Kissell, Kim C. Coley, Alyssa S. Khieu, Elizabeth J. Bunk, Sophia M. C. Herbert, Joni C. Carroll

**Affiliations:** 1 The Hometown Pharmacy, Poland, OH 44514, USA; 2 Department of Pharmacy and Therapeutics, University of Pittsburgh School of Pharmacy, Pittsburgh, PA 15261, USA; 3 Center for Integrative Health, Duquesne University School of Pharmacy, Pittsburgh, PA 15282, USA

**Keywords:** podcasts, social stigma, addiction, pharmacy education, opioid use disorder

## Abstract

The objective of this research was to evaluate the effectiveness of a podcast miniseries to reduce stigma surrounding opioid use disorder (OUD) among student pharmacists. Students in their second and third professional years from two schools of pharmacy listened to five, 10–23 min podcasts incorporated into their coursework. The podcasts highlighted: (1) interviews with OUD professionals and those with lived experiences; (2) types of stigma and how it affects health outcomes; (3) OUD disease state processes, and (4) harm reduction strategies. Surveys assessed changes in perception of OUD and its associated stigma and included free-response and Likert scale questions. Subjects (*n* = 121) who completed a pre- and post-podcast survey were included. Paired *t*-tests assessed changes in survey responses from baseline and a content analysis was performed on all free-responses. There was a statistically significant change from baseline for each survey question, demonstrating a decrease in stigma towards OUD. Free-responses were categorized into four learning domains: (1) Impact of stigma on access to care; (2) Compassion and empathy; (3) Resources and support; and (4) Call to action. Podcasts can be an effective tool to reduce student pharmacist stigma associated with OUD.

## 1. Introduction

The opioid epidemic in the United States (US) has been a public health concern for a large portion of the twenty-first century [1]. In 2020, approximately 2.7 million Americans aged 12 or older had an opioid use disorder (OUD), and overdose death rates rose significantly with the Coronavirus Disease 2019 (COVID-19) pandemic [2]. The 2020 COVID-19 pandemic forced physical distancing, resulting in decreased substance use treatment facility capacities, and interrupted in-person recovery groups and other health care services. This led to decreased patient access to OUD treatment programs while opioid overdose rates increased [3,4,5]. By the end of 2020, The US Centers for Disease Control and Prevention recommended expanding treatment access for substance use disorders, including the use of medications for opioid use disorder (MOUD) [3].

Substance use disorders, including OUD, are highly stigmatized. Stigma is a multidimensional social determinant of health that leads to health inequities and poor health outcomes, including increased morbidity and mortality [5,6]. Goffman defines stigma as “enabling varieties of discrimination that will ultimately deny the individual or group full social acceptance, reduce the individuals’ opportunities, and fuel social inequality” [7]. Barriers, such as stigma associated with OUD and its treatment, are frequently cited by patients as reasons for not seeking treatment and help [8,9,10]. Stigma surrounding OUD occurs at both the structural and individual levels, and the stigma associated with OUD creates more negative perceptions than stigma associated with other health conditions [5,11,12]. The COVID-19 pandemic further revealed the existing structural and individual stigma people with OUD face on an ongoing basis and highlighted a need to provide stigma mitigation training and interventions to more healthcare providers, including pharmacists [5]. A person may also experience societal consequences of stigma, leading to decreases in job opportunities, and the ability to seek and receive healthcare and housing. These societal consequences can further exacerbate health-related problems and outcomes [11,13]. People with OUD may also experience added layers of stigma if they identify with historically marginalized communities, such as those who experienced incarceration or homelessness, those with mental health conditions or disabilities, people of color, people who experience poverty, and individuals from the LGBTQIA+ community, among others [14,15,16].

The stigma associated with OUD continues to be an ongoing problem, even amongst pharmacists and other healthcare providers [9,17,18]. Research has shown that healthcare professionals continue to have judgmental attitudes towards patients affected by OUD [19]. It is imperative for healthcare professionals to help decrease, rather than perpetuate, the stigma associated with OUD [1]. One major way to change the course of the stigma surrounding OUD is to educate healthcare students on why this stigma exists and how to combat it. Addressing stigma can be uncomfortable, or a topic many do not feel adequately prepared to confront. Healthcare professional education currently provides inadequate exposure to stigma associated with OUD [20].

There is a need for improved educational methods to provide stigma mitigation training in the curricula of health profession schools, including pharmacy. Podcasts provide a convenient and flexible way for health science learners to augment classroom, experiential, or traditional didactic learning [21,22,23,24]. Podcasts are audio recordings that frequently utilize storytelling or conversations between individuals to explore a topic [21,25]. Asynchronous learning through podcasts may provide a means of learning about uncomfortable or sensitive subjects, such as stigma, as it allows for students to explore the topic more freely at their own pace and in a comfortable environment [21]. Throughout recent years, podcasts are being used more frequently in educational settings and for health care continuing education [21,26,27,28]. To our knowledge, OUD stigma education delivered via podcasts for students in a professional pharmacy program has not been studied. The objective of this project was to evaluate the effectiveness of a podcast miniseries to reduce stigma surrounding OUD among student pharmacists.

## 2. Materials and Methods

### 2.1. Study Participants

Second-professional year student pharmacists from The University of Pittsburgh School of Pharmacy (*n* = 117) and third-professional year student pharmacists from Duquesne University School of Pharmacy (*n* = 162) who were enrolled in community-based Introductory Pharmacy Practice Experiences (IPPE) or Continuous Professional Development (CPD) courses were eligible for participation. These IPPE and CPD experiences are part of the experiential learning curricula at accredited schools of pharmacy in the US.

### 2.2. Educational Intervention

A podcast miniseries, titled “Let’s Talk Stigma”, consisting of five episodes was developed to address the stigma surrounding OUD [29]. The podcast included audio clips taken from interviews with individuals in long-term recovery, healthcare professional experts, harm reduction specialists, and family and friends of those affected by OUD. Each episode was 10–23 min in length and highlighted various topics on the stigma of OUD. Topics included: (1) the historical context of stigma; (2) treating OUD as a chronic disease state; (3) inter- and intrapersonal stigma, (4) harm reduction and naloxone; and (5) MOUD and their associated stigma.

### 2.3. Study Design and Analysis

Pre-and post-podcast surveys were designed to assess student pharmacists’ stigma associated with OUD. Student pharmacists were required to listen to all five podcast episodes as part of their experiential learning coursework between January and May 2021. Before listening to the podcast miniseries, students were invited to complete a 15-item pre-survey consisting of 5-point Likert scale items that focused on the care of patients with OUD. Students were asked additional demographic questions, and two questions that served as individual identifiers to link pre- and post-survey results. After finishing the final episode of the podcast miniseries, students were invited to complete an optional 17-item post-survey consisting of the same Likert scale items, demographic, identifier questions, and two additional free-response questions. Survey questions are located in Appendix A. For student survey responses to be included in the final analysis, students had to complete both a pre- and post- podcast survey. Each respondents’ pre-and post-survey responses were linked and paired *t*-test analyses were performed. *p* values were reported for each *t*-test, and *p* values ≤ 0.05 were deemed significant. Effect size was calculated using Cohen’s d for paired *t*-tests. A Cohen’s d value of 0.2, 0.5, and 0.8 represents small, medium, and large effect sizes, respectively [30,31]. Subanalyses were conducted for both pre- and post-survey responses for gender identity, prior history of community pharmacy experience, and personal connection with individuals with OUD using independent *t*-tests. There was not enough variability in age and race to perform a subanlysis for these categories. Any unpaired survey responses were excluded from the final statistical analysis. Statistical analyses were performed using IBM^®^ SPSS^®^ Statistics (Version 28; IBM Corp., Armonk, NY, USA). The two post-survey free-response questions asked students the following: (1) What is the most impactful thing you learned from the podcast on reducing opioid use disorder stigma? and (2) How has your attitude toward patients with opioid use disorder changed after listening to the podcast? All free-response data from the post-podcast survey were included in a qualitative content analysis irrespective of whether a student completed a pre-podcast survey. Free-response questions were independently reviewed by three members of the research team (J.C.C., A.S.K., L.K.) using an inductive rapid content analysis method [32,33] First, text data from participants were reviewed line-by-line and grouped by common meanings. These groupings were further categorized into overarching student learning points. A generic inductive approach was used to condense raw text data into a summary format [32]. A series of four study team discussion meetings between three investigators (J.C.C., A.S.K., L.K.) were used to refine and reach consensus on the learning point categories. Quotes that represented the series of student learning points were selected by the investigative team as evidence. Learning points were then organized into four major domains by the research team through consensus discussions.

No incentives were provided to students to participate in this project and participation was voluntary. Surveys were deployed electronically through Qualtrics Online Survey Service (Qualtrics, Provo, UT, USA), and URL links to the survey were provided during regular classroom activities. This research was approved by The University of Pittsburgh Institutional Review Board.

## 3. Results

### 3.1. Quantitative Results

**Figure 1:**
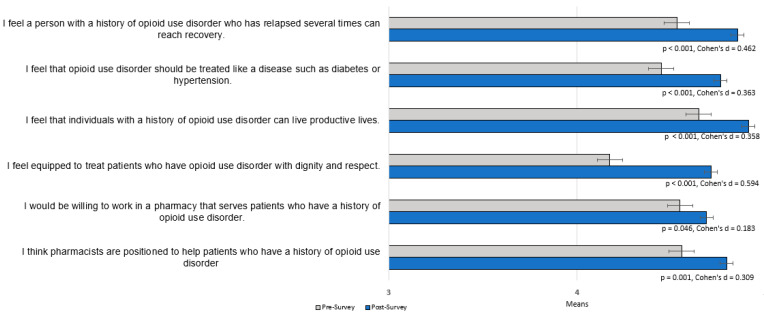
Student pharmacist responses to Likert-style survey questions pre- and post-podcast educational intervention. A response of 1 corresponds to answer choice “strongly disagree,” 5 corresponds to answer choice “strongly agree”.

There were 236 responses (84% response rate) on the pre-survey and 161 responses on the post-survey (58% response rate). Fifty-one percent of students (*n* = 121) completed both a pre- and post-survey which were included in the quantitative data analysis. Student mean age was 22.7 years (range, 21–29 years), a majority of participants identified as women, and most identified as white (Table 1). Approximately 59% of students reported that they personally knew someone who has been affected by a substance use disorder. Results of the paired *t*-test are reported in Figure 1. There was a statistically significant increase in mean response score from baseline for each Likert survey item, representing student agreement with non-stigmatizing statements in the care of patients with OUD. Only question number 3 (see Appendix A for survey questions and Figure 1 for results) had a moderate effect size, while the rest of the Likert questions had a small effect size.

Subanalyses for pre- and post-test survey responses were conducted. Individuals who knew someone with OUD felt more strongly that a person who relapsed could still reach recovery. This was significant only in the post-test survey (*p* = 0.006). Women were more likely to strongly agree that OUD should be treated like a disease. This was significant in both pre- and post-survey responses (*p* = 0.015 and *p* = 0.031, respectively). Students with a history of community pharmacy experience were less likely (*p* = 0.002), on the pre-survey only, to agree with the statement that pharmacists were positioned to help patients who have a history of OUD. This difference was not observed in the post-survey responses after students listened to the podcast. The results of all other subanalyses were not statistically significant.

### 3.2. Qualitative Results

Table 2 outlines four major learning domains and eight specific learning points that emerged from student qualitative survey data. Domains include: (1) Impact of Stigma on Access to Care; (2) Compassion and Empathy; (3) Resources and Support; and (4) Call to Action.

Additionally, many student pharmacists felt hearing stories from individuals affected by OUD through the podcast was an impactful way to learn about stigma. One student remarked, “I think the most impactful aspect was the first-person stories of people’s experiences with substance use disorders and how stigmas have affected them because it gave information from the perspective of those who are directly impacted by the stigma.” Another agreed that “It was powerful to hear peoples’ stories and their experiences. It made me reflect on how I view others suffering with this disorder and reminded me to treat them with the same compassion as everyone else…”. Overall, the podcast enhanced students’ understanding of the impacts of stigma and the influence it has on people’s lives. By hearing stories one student remarked the podcast “... definitely helped humanize those who suffer from this condition”.

## 4. Discussion

The results of this study provide evidence that podcasts may be a useful educational tool for many students to reduce stigma, particularly in experiential learning settings. Podcasts are used as an educational tool in many fields and may foster adult learning [34,35,36]. Our results also provide evidence that podcasts which include stories from individuals with lived experiences may be an effective way to reduce the stigma associated with OUD. Additionally, the four key learning domains that emerged from our results can be applied as a framework in future podcast educational efforts to address stigma surrounding other disease states such as HIV, mental health conditions, and sexually transmitted infections.

With the evolution of the COVID-19 pandemic, institutions of education are utilizing less-traditional educational tools to provide students the highest quality of education possible [37,38]. For the majority of the 2020–2021 academic year, classes were conducted virtually using various online platforms. In the present study, this permitted the use of technology for students and educators to conduct asynchronous learning and allowed students to learn about stigma in an environment of their choosing. Our results demonstrated that the podcast enhanced student learning that was occurring during an experiential education experience. Previous research has shown that podcasts may be a preferred method of learning for health sciences trainees [39,40]. Incorporating a variety of learning tools facilitates student engagement in asynchronous learning and provides flexibility for learners [41]. Podcasts may become more useful as institutions of learning continue to incorporate asynchronous learning into their curricula.

Our podcast episodes were designed to be short in length, so that students or practitioners could listen to them during their daily commutes to experiential learning or work sites. Other research on podcast length also shows that podcasts of 30 min or less are preferred [40,42]. We purposely incorporated the podcasts into the experiential learning curricula at both schools of pharmacy within this study. This allowed the podcast to be quickly implemented into the Schools’ curricula alongside a practice-based educational experience. Utilizing podcasts as a form of asynchronous learning shows promise particularly in the setting of experiential education, where asynchronous content complements on-site learning. This structure allowed students to immediately apply ideas they learned from the podcasts into practice. Our podcasts are currently available at no cost for pharmacist and pharmacy technician continuing education credit and through pharmacy podcast media outlets and can be utilized by others in their curricula (Appendix B) [29,43].

Many student pharmacists have work experience in community pharmacies while others get exposure to community pharmacy practice through experiential education. Our data suggests that students with experience working in community pharmacy were less likely, on the pre-survey only, to agree with the statement that pharmacists were positioned to help patients who have a history of OUD. This difference was not observed in the post-survey responses. One explanation is that student exposure to the workload of community pharmacy combined with insufficient community pharmacy staffing, especially during the COVID-19 pandemic, may have influenced their perceptions of pharmacists’ ability to provide OUD services to their patients. This result also suggests that the podcast, combined with their experiential learning experiences at pharmacy sites, may have helped students recognize how community pharmacists could serve these patients in light of their limited resources.

Another interesting finding was respondents who identified as women were more likely to agree that OUD should be treated like a chronic disease. Other Pennsylvania OUD stigma survey data suggests that more than half of respondents to a statewide public survey agree that OUD is a medical illness [44]. Given that our survey was conducted in health sciences professional programs, it is not surprising that most of our survey respondents felt that OUD should be treated like a disease. The data in the literature around sex and gender differences related to stigma is mixed [11,44,45]. A recent study in Pennsylvania found men were more likely than women to hold stigmatizing attitudes [44]. Howick and colleagues conducted a meta-analysis of studies where patients rated their practitioners’ empathy and compared studies with mostly female versus male practitioners [46]. These authors found that studies including predominately female practitioners had significantly higher empathy scores. Further research on how gender influences empathy expression regarding OUD would help clarify these findings in our study.

Podcasts often incorporate storytelling into their format. Storytelling and personal testimonies are techniques that can be used to combat stigma [35,47]. One important aspect of our podcast miniseries was the incorporation of testimonials from persons with lived experiences who experienced stigma related to their OUD. We believe that it was important to include multiple individuals’ journeys and perspectives in one episode. Students remarked specifically how hearing from individuals with lived experience had an impact on them personally and on their stigma around OUD. Our post-survey data showed that students who knew someone with OUD felt more strongly that a person who relapsed could still reach recovery. Previous research showed that individuals having a personal experience with opioid misuse or OUD was associated with lower levels of stigma, however having a family member or friend with OUD was not necessarily correlated with lower levels of stigma [48,49]. One possible reason for this difference in findings in the present study is that our survey was conducted among health sciences students who received training on compassion and empathy in other therapeutic areas. Our use of storytelling, however, aligns with previous research on teaching about stigma surrounding mental health conditions using lived experiences and testimonials in the health sciences [47,50]. The stories shared from a variety of people during these podcasts allowed students to hear about how stigma associated with OUD impacts the daily lives of individuals and how they can help reduce stigma amongst the healthcare community. Sharing personal testimonies and stories, reframing the stigmatized condition, and introducing resources have been shown to be more successful at reducing stigma when they are presented in tandem with each other compared to alone [51]. This concept aligns with the four learning domains we uncovered from our qualitative analysis.

### Limitations

There are several limitations to this study. Students were in the midst of their didactic curriculum at their respective college of pharmacy during the time of this study. Students could have had confounding education or life experiences that contributed to their reduction of stigma. Students may have had personal experiences that could have changed their perception of stigma with OUD that was unrelated to the podcast. Additionally, the presence or absence of stigma, in general, is a difficult marker to capture and quantify and may change over time with life experiences and social environments.

One limitation of podcast use itself is that podcasts are a form of auditory learning and may not be appropriate or accessible for every learner. To make these more accessible, we suggest written transcripts are also included alongside the podcasts for learners.

## 5. Conclusions

The results of this study show that podcasts can be utilized as an effective method to help lower the stigma associated with OUD among student pharmacists. Podcasts can give students a unique learning experience and can allow for asynchronous learning on topics that may be more sensitive.

## Figures and Tables

**Table 1:** Student pharmacist respondent demographics (*n* = 121).

Respondent Characteristics	Value
Gender Identity, *n*	
-Woman	92 (70.6%)
-Man	28 (23.1%)
-Non-binary	1 (0.8%)
Race, *n*	
-White	104 (86%)
-Asian/Pacific Islander	12 (9.9%)
-Black/African American	1 (0.8%)
-Other	4 (3.3%)
College of Pharmacy, *n*	
-Duquesne University	87 (71.9%)
-University of Pittsburgh	34 (28.1%)
Know someone personally who has a substance use disorder, *n*	
-Yes	71 (58.7%)
-No	50 (41.3%)
Mean Age, Years (SD)	22.7 (1.2)

**Table 2:** Student learning points from podcast.

Learning Domains	Student Learning Points	Student Quotes
**Impact of Stigma on Access to Care** This domain teaches the learner about the impact stigma can have on access to care.	People with OUD regularly experience stigma and it impacts their lives in many ways	“I learned how patients with opioid use disorder can be stigmatized so often in their everyday lives and just how much of a struggle it can be to deal with that on top of their disorder.” “I learned how the stigma can truly affect the patient and their families in a way much bigger than just hurting one’s feelings.” “I thought one of the more poignant points was the discussion about interpersonal stigma. To hear these individuals talk about the ways they had discounted themselves was truly heartbreaking and certainly opened my eyes to the hardships they experience.”
	2. Stigma may prevent patients from seeking care for recovery	“The most impactful thing I learned was the true struggles people have in not only overcoming the disorder but facing the stigma when they have chosen the positive step to recovery. Sometimes even when searching for help, when the proper support and encouragement are not available, it can discourage the road to recovery. “ “My attitude has not changed much but I am more aware of how actions that may seem small or insignificant to me can drastically affect the treatment these patients seek or receive.”
**Compassion and Empathy** This domain teaches the learner the importance of providing care with compassion and empathy.	3. Improved empathy and understanding of the complexity of OUD is important	“I now am more empathetic of the difficulties of the disease, particularly getting access to the help they want and need.” “I learned how important it is to treat these patients with compassion. There is so much stigma out there, and by being compassionate and non-stigmatizing towards our patients it can positively impact their recovery.”
	4. Pharmacists have a responsibility to treat patients with OUD with compassion and respect, to help reduce stigma	“This podcast has made me have a better understanding of opioid use disorder and that pharmacists play a very essential role in these patient’s care. Since we are the most accessible and also the ones who are dispensing these medications we must be compassionate and respectful to ensure they receive the best care.” “As pharmacists, we will encounter a lot of people with this disease state so it is important that we can educate them and help them get the appropriate medication in order to save their lives.” “After listening to the podcast episodes, the most impactful thing to me as a pharmacy student was wanting to be the healthcare professional that provides patients with positive experiences and makes them feel like they deserve to have their OUD treated. Listening to stories of negative encounters with healthcare providers as people are going through recovery had an impact on me, and inspired me to be a better example of our profession and do better for this patient population.” “...sometimes the patient just needs someone to listen and be there for them as they don’t want to have a relapse and access to their medication is an important part of that.”
**Resources and Support** This domain outlines examples of resources and support that the learner can provide to those with a specific stigmatized condition.	5. Harm reduction strategies are important to help people into recovery	“The most impactful thing I learned is how [naloxone] is so helpful and a life saving thing for opioid overdose patients. Pharmacists should educate and encourage everyone [to] carry [naloxone] with them.” “The most impactful thing that I learned was that providing clean syringes and needles are not only helpful to prevent the spread of disease but it can also keep the patient alive and healthy until they are ready to seek treatment for their condition. I think this is a really important point and is something that could change people’s minds who are hesitant to sell [syringes].” “We need to focus more on harm reduction versus just trying to place more restrictions, restrictions will only continue to worsen the stigma on opioid use disorder.”
	6. Medications for opioid use disorder are helpful to patients but are also stigmatized	“I never thought about how a patient on [medications for opioid use disorder] may be stigmatized by other people with OUD on top of the stigma they receive from friends, family, and health care professionals. It gives me an added level of empathy for them and makes me want to compensate for the negative reactions of others by showing them my acceptance as whole-heartedly as I can.” “I learned that it can be very stressful for patients to sometimes pick up their medications for treatment for their opioid disorder and that they can feel judged or looked down on…” “...there are barriers that prevent people with OUD from accessing medications to help them fight [opioid use disorder].” “…It was also helpful learning more about the [medications for opioid use disorder] and how they can reduce the chance of fatal overdoses by 50%.”
**Call to Action** This domain provides a call to action for the learner by describing what the learner should do as a result of the educational content.	7. OUD should be viewed and treated as a chronic disease	“My biggest learning point is how substance use disorder should be considered as a disease, and can be likened to diabetes. I never really thought about it this way.” “Understanding that it alters the brain and is a chronic relapsing disease. It does not take accountability away from the patient, but it does reduce stigma when addiction is reinforced as a disease.” “After listening to the podcasts, I now view opioid use disorder as more of a disease state than a choice. I now feel passionately that these individuals should be treated with the same empathy and care as if they had a condition like diabetes or hypertension.”
	8. The language used regarding OUD matters	“The most important thing I have learned about reducing stigma is to watch my language. Even if something I say feels neutral to me, it can have a big impact on the patient’s perception of me and even their confidence in themselves.” “How important it is to use the correct language especially if a patient refers to themselves as a “junkie” or “druggie” etc. It is important to show the patient that healthcare providers do not see them as that, but as an individual with a disease that needs treatment like all our other patients.” “When learning about opioid use disorders, I learned how to use appropriate words when talking to a patient about their substance use disorder to make sure they feel supported and do not feel judged or looked down on.”

## Data Availability

The data presented in this study are available on request from the corresponding author.

## Appendix A. Survey Questions

This research is being conducted by the University of Pittsburgh. The purpose of this research study is to gather student pharmacist perspectives about stigma associated with opioid-use disorder and opinions on an educational training program about this topic.

We are inviting student pharmacists from the University of Pittsburgh and Duquesne University Schools of Pharmacy to complete this brief (approximately 5–10 min) survey. There are no foreseeable risks associated with this research, nor are there any direct benefits to you. All survey responses are confidential, and results will be kept under lock and key. Your participation is voluntary, and you may choose to withdraw from participation in the survey at any time. This study is being conducted by Joni Carroll, PharmD who can be reached at (412)383-4415, if you have any questions.

Q1 I think pharmacists are positioned to help patients who have a history of opioid use disorder.

□ Strongly agree (1)
□ Somewhat agree (2)
□ Neither agree nor disagree (3)
□ Somewhat disagree (4)
□ Strongly disagree (5)

Q2 I would be willing to work in a pharmacy that serves patients who have a history of opioid use disorder.

□ Strongly agree (1)
□ Somewhat agree (2)
□ Neither agree nor disagree (3)
□ Somewhat disagree (4)
□ Strongly disagree (5)

Q3 I feel equipped to treat patients who have opioid use disorder with dignity and respect.

□ Strongly agree (1)
□ Somewhat agree (2)
□ Neither agree nor disagree (3)
□ Somewhat disagree (4)
□ Strongly disagree (5)

Q4 I feel that individuals with a history of opioid use disorder can live productive lives.

□ Strongly agree (1)
□ Somewhat agree (2)
□ Neither agree nor disagree (3)
□ Somewhat disagree (4)
□ Strongly disagree (5)

Q5 I feel that opioid use disorder should be treated like a disease such as diabetes or hypertension.

□ Strongly agree (1)
□ Somewhat agree (2)
□ Neither agree nor disagree (3)
□ Somewhat disagree (4)
□ Strongly disagree (5)

Q6 I feel a person with a history of opioid use disorder who has relapsed several times can reach recovery.

□ Strongly agree (1)
□ Somewhat agree (2)
□ Neither agree nor disagree (3)
□ Somewhat disagree (4)
□ Strongly disagree (5)

Q7 What is the most impactful thing you learned from the podcast on reducing opioid use disorder stigma?

________________________________________________________________
________________________________________________________________

Q8 How has your attitude toward patients with opioid use disorder changed after listening to the podcast?

________________________________________________________________
________________________________________________________________

Q9 What is your age?

________________________________________________________________

Q10 What best describes your gender? (Select as many as apply)

□ Man
□ Woman
□ Non-binary
□ Cisgender
□ Transgender

Q11 What best describes your race/ethnicity? (Select all that apply)

□ Asian/Pacific Islander
□ Black/African American
□ Hispanic/Latino
□ Native American/American Indian
□ White
□ Other ________________________________________________

Q12 In what pharmacy settings have you currently/previously worked or volunteered? (select all that apply)

□ Community Pharmacy
□ Hospital Pharmacy
□ Specialty Pharmacy
□ Long-Term Care Pharmacy
□ Managed Care
□ Other ________________________________________________
□ No Pharmacy Work Experience

Q13 What pharmacy school do you attend?

○ University of Pittsburgh School of Pharmacy
○ Duquesne University School of Pharmacy

Q14 In what year will you graduate from pharmacy school?

○ 2024
○ 2023
○ 2022
○ 2021

Q15 I personally know someone who has been affected by substance use disorder or drug use.

○ Yes
○ No

The last two questions will allow the research team to link your before and after answers without identifying you personally.

Q16 What is your birth month?

○ January
○ February
○ March
○ April
○ May
○ June
○ July
○ August
○ September
○ October
○ November
○ December

Q17 What are the last three (3) digits of your phone number?

________________________________________________________________

## Appendix B

The “Let’s Talk Stigma” podcast series is published by the Pharmacy Podcast Network. Episodes are available by searching “Let’s Talk Stigma” on most major podcast vendors (e.g., Spotify, Apple Podcasts). The podcast is also available for free continuing education credit for pharmacists and pharmacy technicians through the Pennsylvania Pharmacists Association.

Available at: https://www.papharmacists.com/page/StigmaPodcast (accessed on 21 November 2022) and https://pharmacypodcast.com/2022/03/17/lets-talk-stigma/ (accessed on 21 November 2022).

## References

[1] Lyden J., Binswanger I.A. (2019). The United States opioid epidemic. Semin. Perinatol..

[2] Substance Abuse Center for Behavioral Health Statistics and Quality (2022). Results from the 2020 National Survey on Drug Use and Health: Detailed Tables.

[3] Centers for Disease Control and Prevention Emergency Preparedness and Response. Increase in Fatal Drug Overdoses across the United States Driven by Synthetic Opioids before and during the COVID-19 Pandemic. https://emergency.cdc.gov/han/2020/han00438.asp.

[4] Meadowcroft D., Davis W. (2022). Understanding the Effect of the COVID-19 Pandemic on Substance Use Disorder Treatment Facility Operations and Patient Success: Evidence from Mississippi. Subst. Abus. Res. Treat..

[5] Murthy P., Narasimha V.L. (2021). Effects of the COVID-19 pandemic and lockdown on alcohol use disorders and complications. Curr. Opin. Psychiatry.

[6] Hatzenbuehler M.L., Phelan J.C., Link B.G. (2013). Stigma as a Fundamental Cause of Population Health Inequalities. Am. J. Public Health.

[7] Goffman E. (1963). Stigma: Notes on the Management of Spoiled Identity.

[8] Goodyear K., Haass-Koffler C.L., Chavanne D. (2018). Opioid use and stigma: The role of gender, language and precipitating events. Drug Alcohol Depend..

[9] Tsai A.C., Kiang M.V., Barnett M.L., Beletsky L., Keyes K.M., McGinty E.E., Smith L.R., Strathdee S.A., Wakeman S.E., Venkataramani A.S. (2019). Stigma as a fundamental hindrance to the United States opioid overdose crisis response. PLoS Med..

[10] Witte T.H., Jaiswal J., Mumba M.N., Mugoya G.C.T. (2021). Stigma Surrounding the Use of Medically Assisted Treatment for Opioid Use Disorder. Subst. Use Misuse.

[11] Kennedy-Hendricks A., Barry C.L., Gollust S.E., Ensminger M.E., Chisolm M.S., McGinty E.E. (2017). Social Stigma toward Persons with Prescription Opioid Use Disorder: Associations with Public Support for Punitive and Public Health–Oriented Policies. Psychiatr. Serv..

[12] Corrigan P.W., Kuwabara S.A., O’Shaughnessy J. (2009). The public stigma of mental illness and drug addiction: Findings from a stratified random sample. J. Soc. Work..

[13] Singh S., Kumar S., Sarkar S., Balhara Y.P.S. (2018). Quality of Life and its Relationship with Perceived Stigma among Opioid Use Disorder Patients: An Exploratory Study. Indian J. Psychol. Med..

[14] Substance Abuse and Mental Health Services Administration (2020). The Opioid Crisis and the Black/African American Population: An Urgent Issue.

[15] Kreek M.J. (2011). Extreme marginalization: Addiction and other mental health disorders, stigma, and imprisonment. Ann. N. Y. Acad. Sci..

[16] Paschen-Wolff M.M., Velasquez R., Aydinoglo N., Campbell A.N.C. (2022). Simulating the experience of searching for LGBTQ-specific opioid use disorder treatment in the United States. J. Subst. Abus. Treat..

[17] Hall E.A., Cernasev A., Nasritdinova U., Veve M.P., Hohmeier K.C. (2020). Stigma of Opioid Use Disorder and Its Indirect Effects on Student Pharmacists’ Perceptions and Attitudes. Pharmacy.

[18] Burgess A., Bauer E., Gallagher S., Karstens B., Lavoie L., Ahrens K., O’Connor A. (2021). Experiences of stigma among individuals in recovery from opioid use disorder in a rural setting: A qualitative analysis. J. Subst. Abus. Treat..

[19] Salmond S., Allread V. (2019). A Population Health Approach to America’s Opioid Epidemic. Orthop. Nurs..

[20] Volkow N.D., Blanco C. (2021). The changing opioid crisis: Development, challenges and opportunities. Mol. Psychiatry.

[21] Malka R., Villwock J., Faucett E.A., Bowe S. (2021). Podcast-based learning in otolaryngology: Availability, breadth, and comparison with other specialties. Laryngoscope.

[22] Miesner A.R., Lyons W., McLoughlin A. (2017). Educating medical residents through podcasts developed by PharmD students. Curr. Pharm. Teach. Learn..

[23] De Gagne J.C., Park H.K., Hall K., Woodward A., Yamane S., Kim S.S. (2019). Microlearning in health professions education: Scoping review. JMIR Med. Educ..

[24] Kelly J.M., Perseghin A., Dow A.W., Trivedi S.P., Rodman A., Berk J. (2022). Learning through Listening: A Scoping Review of Podcast Use in Medical Education. Acad. Med..

[25] Zumach G., Portillo E. (2020). Cultivating a Community of Practice through Podcasting. Innov. Pharm..

[26] Bolliger D.U., Supanakorn S., Boggs C. (2010). Impact of podcasting on student motivation in the online learning environment. Comput. Educ..

[27] Back D.A., von Malotky J., Sostmann K., Hube R., Peters H., Hoff E. (2017). Superior Gain in Knowledge by Podcasts Versus Text-Based Learning in Teaching Orthopedics: A Randomized Controlled Trial. J. Surg. Educ..

[28] National STD Curriculum Podcast University of Washington. https://www.std.uw.edu/podcast.

[29] Beyond the Sig Podcast—Let’s Talk Stigma Opioid Podcast Miniseries. https://www.papharmacists.com/page/StigmaPodcast.

[30] Cohen J. (1988). Statistical Power Analysis for the Behavior Sciences.

[31] Cumming G. (2012). Understanding the New Statistics: Effect Sizes, Confidence Intervals, and Meta-Analysis.

[32] Kleinheksel A.J., Rockich-Winston N., Tawfik H., Wyatt T.R. (2020). Demystifying Content Analysis. Am. J. Pharm. Educ..

[33] Patton M.Q. (2015). Qualitative Research & Evaluation Methods: Integrating Theory and Practice.

[34] McNamara S.W., Shaw M., Wilson K., Cox A. (2021). Educational Podcasts in Kinesiology: A Scoping Review. Kinesiol. Rev..

[35] Davidson S.M., Grunau Z., Marcovitz D., Gerdner O.A., Stoklosa J., Vestal H.S. (2019). Narrative Podcasts as a Teaching Tool in Psychiatry. Acad. Psychiatry.

[36] Popova A., Edirisingha P. (2010). How can podcasts support engaging students in learning activities?. Procedia-Soc. Behav. Sci..

[37] Sandhu P., de Wolf M. (2020). The impact of COVID-19 on the undergraduate medical curriculum. Med. Educ. Online.

[38] Ahmady S., Kallestrup P., Sadoughi M.M., Katibeh M., Kalantarion M., Amini M., Khajeali N. (2021). Distance learning strategies in medical education during COVID-19: A systematic review. J. Educ. Health Promot..

[39] Mallin M., Schlein S., Doctor S., Stroud S., Dawson M., Fix M. (2014). A Survey of the Current Utilization of Asynchronous Education among Emergency Medicine Residents in the United States. Acad. Med..

[40] Chin A., Helman A., Chan T. (2017). Podcast Use in Undergraduate Medical Education. Cureus.

[41] Brady A.K., Pradhan D. (2020). Learning without Borders: Asynchronous and Distance Learning in the Age of COVID-19 and Beyond. ATS Sch..

[42] Riddell J., Swaminathan A., Lee M., Mohamed A., Rogers R., Rezaie S.R. (2017). A Survey of Emergency Medicine Residents’ Use of Educational Podcasts. West. J. Emerg. Med..

[43] Let’s Talk Stigma. Pharmacy Podcast Network. Welcome to the Pharmacy Podcast Network. https://pharmacypodcast.com/2022/03/17/lets-talk-stigma/#:~:text=The%20Let’s%20Talk%20Stigma%20podcast,individuals%20with%20opioid%20use%20disorders.

[44] Kaynak Ö., Whipple C.R., Bonnevie E., Grossman J.A., Saylor E.M., Stefanko M., McKeon C., Smyser J., Kensinger W.S. (2022). The Opioid Epidemic and the State of Stigma: A Pennsylvania Statewide Survey. Subst. Use Misuse.

[45] Adams Z.W., Taylor B.G., Flanagan E., Kwon E., Johnson-Kwochka A.V., Elkington K.S., Becan J.E., Aalsma M.C. (2021). Opioid Use Disorder Stigma, Discrimination, and Policy Attitudes in a National Sample of U.S. Young Adults. J. Adolesc. Health.

[46] Howick J., Steinkopf L., Ulyte A., Roberts N., Meissner K. (2017). How empathic is your healthcare practitioner? A systematic review and meta-analysis of patient surveys. BMC Med. Educ..

[47] Kassam A., Glozier N., Leese M., Loughran J., Thornicroft G. (2011). A controlled trial of mental illness related stigma training for medical students. BMC Med. Educ..

[48] Lin Q., Kolak M., Watts B., Anselin L., Pollack H., Schneider J., Taylor B. (2022). Individual, interpersonal, and neighborhood measures associated with opioid use stigma: Evidence from a nationally representative survey. Soc. Sci. Med..

[49] Taylor B.G., Lamuda P.A., Flanagan E., Watts E., Pollack H., Schneider J. (2021). Social Stigma toward Persons with Opioid Use Disorder: Results from a Nationally Representative Survey of U.S. Adults. Subst. Use Misuse.

[50] Patten S.B., Remillard A., Phillips L., Modgill G., Szeto A.C., Kassam A., Gardner D.M. (2012). Effectiveness of contact-based education for reducing mental illness-related stigma in pharmacy students. BMC Med. Educ..

[51] Knaak S., Modgill G., Patten S.B. (2014). Key Ingredients of Anti-Stigma Programs for Health Care Providers: A Data Synthesis of Evaluative Studies. Can. J. Psychiatry.

